# Mitral valve replacement via right thoracotomy approach for prevention of mediastinitis in a female patient with long-term uncontrolled diabetes mellitus: a case report

**DOI:** 10.1186/1749-8090-5-38

**Published:** 2010-05-17

**Authors:** Naoto Fukunaga, Takashi Hashimoto, Yasuhisa Ozu, Shigeru Komori, Yu Shomura, Hiroshi Fujiwara, Michihiro Nasu, Yukikatsu Okada

**Affiliations:** 1 Department of Cardiovascular surgery, Kobe City Medical Center General Hospital, 4-6 Minatojimanakamachi, Chuo-ku, Kobe, Hyogo 650-0046, Japan

## Abstract

A 76-year-old woman with a history of percutaneous transvenous mitral commissurotomy and repeated hospital admissions due to heart failure was referred for an operation for severe mitral valve stenosis. She presented with hypertension, hyperlipidemia and cerebral infarction with stenosis of right internal carotid artery, retinopathy, neuropathy and nephropathy caused by long-term uncontrolled diabetes mellitus, hemoglobin A1c of 9.4%, and New York Heart Association (NYHA) functional classification of 3/4. Echocardiography revealed severe mitral valve stenosis with mitral valve area of 0.6 cm^2^, moderate tricuspid valve regurgitation, and dilatation of the left atrium. Taking into consideration the NYHA functional classification and severe mitral valve stenosis, an immediate surgical intervention designed to prevent mediastinitis was performed. The approach was via the right 4th thoracotomy, as conventional sternotomy would raise the risk of mediastinitis. Postoperative antibiotics were administered intravenously for 2 days, and signs of infection were not recognized.

In patients with long-term uncontrolled diabetes mellitus, mid-line sternotomy can easily cause mediastinitis. The choice of operative approach plays an important role in preventing this complication. In this report, the importance of the conventional right thoracotomy for prevention for mediastinitis is reviewed.

## Background

In patients with long-term uncontrolled diabetes mellitus, mediastinitis is a critical complication of cardiovascular surgery and may easily be caused by mid-line sternotomy. Therefore, the choice of operative approach plays an important role in preventing mediastinitis. In this report, the importance of a minimally invasive conventional right thoracotomy approach for the prevention of mediastinitis is reviewed.

## Case report

A 76-year-old woman with a history of percutaneous transvenous mitral commissurotomy and repeated admissions due to heart failure was referred for the purpose of an operation for severe mitral valve stenosis. The patient had dyspnea, retinopathy, neuropathy and nephropathy caused by long-term uncontrolled diabetes mellitus, Basedow's disease, hypertension, hyperlipidemia and cerebral infarction with stenosis of the right internal carotid artery, and New York Heart Association (NYHA) functional classification of 3/4. Laboratory examination revealed plasma creatinine of 1.06 mg/dl and a hemoglobin A1c of 9.4%. Transthoracic echocardiography revealed severe mitral valve stenosis with a mitral valve area of 0.6 cm^2^, moderate tricuspid valve regurgitation, and dilatation of the left atrium. The patient had previously delayed a mitral valve operation because of uncontrolled diabetes mellitus. Taking into consideration the NYHA functional classification and symptoms associated with severe mitral valve stenosis, an immediate operation was performed. Postoperative infection in the context of uncontrolled diabetes mellitus was a major concern. As conventional sternotomy would raise the risk of mediastinitis, the right thoracotomy approach was chosen to prevent mediastinitis by avoiding the splitting the sternum.

Through the right 4th thoracotomy approach, cardiopulmonary bypass was instituted by placing two venous cannulas into the superior and inferior vena cava, and one arterial cannula into the right femoral artery. Once on cardiopulmonary bypass, systemic temperature was dropped, ascending aorta was cross-clamped and the heart was arrested by retrograde perfusion of cold blood cardioplegia. Mitral valve replacement with a prosthetic valve (Mosaic Ultra Porcine Valve, 27 mm) and tricuspid annuloplasty with a prosthetic ring (Duran Ancore Annuloplasty Band, 27 mm) were performed, and perioperative prophylactic intravenous vancomycin (1 g) was administered under differential lung ventilation. Postoperatively, cefazolon sodium (2 × 1 g per day) was administered intravenously for 2 days. Postoperative transthoracic echocardiography revealed mild mitral valve regurgitation and, mild pericardial effusion, and an ejection fraction of 68%. In addition to improvement of clinical data, the patient was able to walk without any complaints, indicating NYHA classification of 1/4.

**Figure 1:**
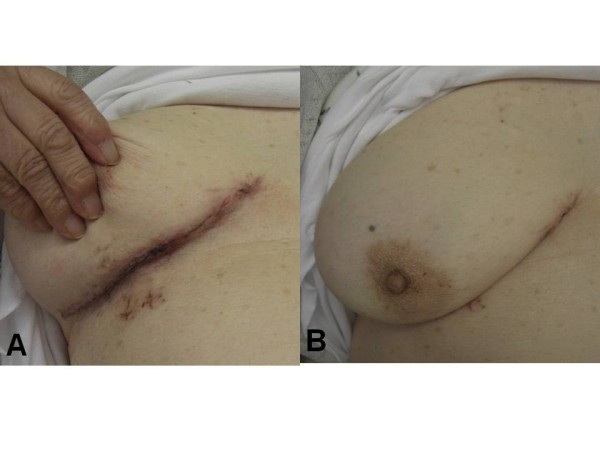
**Postoperative photograph of wound on day 10 after surgery**. (a) Signs of infection are not visible. (b) Incision line is nearly concealed by the right breast.

Postoperative infectious signs were not recognized (Figure 1a and 1b), and the patient was discharged on day 14 after surgery.

## Discussion

Minimally invasive mitral valve surgical approaches consist of partial sternotomy, right parasternotomy, right thoracotomy, and left thoracotomy. With improvement of perioheral perfusion systems, use of these approaches is expanding all over the world. In some institutions, these approaches are standard surgical approaches for valve surgery. The 30-day mortality rate, major complications such as renal failure and neurological deficits, and 5-year overall survival for these approaches for mitral valve are satisfactory. The advantages of these minimally invasive surgical approaches include the avoidance of sternal division, preservation of sternal stability, reduced blood loss and transfusions, reduced infection and hospitalizations, and avoidance of visible scarring. Disadvantages include difficulty in exposing the atrium, ventricle and mitral valves, increased distance to mitral valve, and operation time [1,2].

Sternal wound infection, either superficial or deep, are the most significant postoperative complications in cardiovascular surgery. The latter, namely mediastinitis, can invade bone, muscle and the retroperitoneal space and subsequently result in critical deterioration. The rate of occurrence ranges from 1 to 2% [3]. Staphylococcus aureus and Streptococcus epidermidis account for 70 to 80% of these infections.

Risk factors for sternal dehiscence or wound infection include diabetes mellitus, age > 75 years, chronic obstructive pulmonary disease, obesity, congestive heart failure, peripheral vascular disease, and sternal instability [3,4]. In a study conducted by The Society of Thoracic Surgeons, mediastinitis accounted for a quarter of patients with major infections, and the most common clinical predictors associated with mediastinitis were a body mass index of 30 to 40 kg/m^2 ^[2], diabetes mellitus, previous myocardial infarction, urgent operative status and hypertension [5].

Another report found that risk factors for postoperative mediastinitis include female gender, age > 70 years, diabetes mellitus, and methicillin-resistant Staphylococcus aureus [6].

The mainstays for prevention of mediastinitis are recognition of risk factors in patients, preoperative or intraoperative prophylatic antibiotics, and control of blood glucose concentration. Additionally, the operative approach plays an important role in preventing mediastinitis. In contrast to mid-line full sternotomy, mediastinitis has not been recognized with minimally invasive approaches [7]. Because the thoracotomy approach does not require sternal division and preserve sterna stability, it may reduce the rate of infection.

The present patient had three of the conventional risk factors associated with mediastinitis: diabetes mellitus, female gender, and old age. Additionally, according to the analysis by Gummert et al. [7], mid-line full sternotomy was counter-indicated in this patient. In view of the risk factors, a surgical approach considered more suitable for patients at risk for mediastinitis was selected. During the perioperative and postoperative courses, prophylactic antibiotics and control of blood glucose concentration were also used to prevent mediastinitis.

## Competing interests

The authors declare that they have no competing interests.

## Authors' contributions

NF wrote this manuscript and revised it.

NF, TH, YO, SK, YS, HF, MN and YO performed the operation and recommended me to write this case and advised me to revise it. All authors read and approved the final manuscript.

## Consent

Written informed consent was obtained from the patient for publication of this case report and any accompanying images. A copy of the written consent is available for review by the Editor-in-Chief of this journal.
